# The effect of smoking on caries-related microorganisms

**DOI:** 10.18332/tid/105913

**Published:** 2019-04-18

**Authors:** Jiayi Wu, Mingyun Li, Ruijie Huang

**Affiliations:** 1 Department of Endodontic Dentistry, State Key Laboratory of Oral Diseases, National Clinical Research Center for Oral Diseases, West China Hospital of Stomatology, Sichuan University, Chengdu, China; 2 State Key Laboratory of Oral Diseases, National Clinical Research Center for Oral Diseases, West China Hospital of Stomatology, Sichuan University, Chengdu, China; 3 Department of Pediatric Dentistry, West China Hospital of Stomatology, Sichuan University, Chengdu, China

**Keywords:** smoking, dental caries, nicotine, bacterial virulence factors

## Abstract

**INTRODUCTION:**

Epidemiological studies have shown a close relationship between smoking and dental caries. Bacteria are one of the essential factors of caries formation. The imbalance of cariogenic bacteria and commensal bacteria in dental plaque results in higher production of acid that can corrode dental hard tissue. The aim of our review is to summarize the effect of smoking on caries-related bacteria.

**METHODS:**

English articles available in Pubmed and ScienceDirect databases and published before December 2018 were searched. A variety of evidence was collected including not only the influence of cigarette products on bacteria strains *in vitro* but also their effect on bacterial composition in saliva and dental plaque *in vivo*. We particularly emphasize the mechanisms by which nicotine acts on oral bacteria.

**RESULTS:**

The components of cigarettes promote the growth of cariogenic microorganisms. The mechanisms of how nicotine enhances *Streptococcus mutans, Lactobacilli, Streptococcus gordonii, Actinomyces* and *Candida albicans* are described separately in detail. The commensal bacteria, *Streptococcus sanguinis*, show less competitive capability in the presence of nicotine. Smoking influences saliva by lowering the buffer capability, altering its chemical agent and bacterial components, and therefore promotes the formation of a caries-susceptible environment.

**CONCLUSIONS:**

Cigarette smoking and nicotine exposure promote the cariogenic activity of oral microorganisms and the formation of a caries-susceptible environment. This suggests that smokers should quit smoking, amongst other health reasons, also for their oral health.

## INTRODUCTION

Numerous epidemiological studies around the world have reported a close relationship between smoking and the occurrence of dental caries. In Italy, smoking military personnel (including 94.6% men and 5.4% women) have a higher decayed, missing, filled teeth (DMFT) score than non-smoking personnel^1^. In Finland, daily smoking has been found to increase 4-year caries experience in adults^2^. A study in Scotland has shown that if a pregnant woman smokes it might result in her child having a higher prevalence of caries than a child born to a non-smoking mother^3^. In Portugal, smoking has been confirmed as a risk factor for dental caries, and avoiding exposure to smoking leads to a 7% decrease in caries incidence^4^. A systematic review by Benedetti et al.^5^ also verified that tobacco smoking had a close association with an increased risk of caries. However, there were differences between smokers and non-smokers in regard to personal education and economic situation^6^. Investigation showed that smokers tended to have bad eating habits, payed less attention to oral self-care, rarely seeking professional medical treatment and had poor compliance after treatment^7-9^. All these behaviors^6-9^ mentioned above could increase the incidence of caries. Further evidence to verify the cariogenic mechanisms of smoking needs to be researched. Nowadays, researchers have found that smoking has an effect on caries-related bacteria.

## METHODS

Articles in English that were available in the Pubmed and ScienceDirect databases and published before December 2018 were searched. We collected and summarized the evidence of smoking and cigarette products not only as to how they influence the growth and metabolism of caries-related bacteria *in vitro* but also how they affect the saliva and dental plaque *in vivo*.

## RESULTS

The components of cigarettes promote the growth of cariogenic microorganisms. The mechanisms of how nicotine enhances *Streptococcus mutans, Lactobacilli, Streptococcus gordonii, Actinomyces* and *Candida albicans* are described separately in detail. The commensal bacteria, *Streptococcus sanguinis*, show less competitive capability in the presence of nicotine. Smoking influences saliva by lowering the buffer capability, altering its chemical agent and bacterial components, therefore promoting the formation of a caries-susceptible environment.

### The influence of cigarette products on the growth of oral bacteria

It is well known that bacteria create the preconditions for caries. Bacteria in the oral cavity produce acid by degrading the fermentable carbohydrates through the secretion of enzymes or metabolism, so as to induce further demineralization of dental hard tissues^10^. An early study^11^ in 1991 claimed that smoking inhibited the growth of gram-positive cocci including *Neisseria*, one of the early colonizers in dental plaque. Smokers were considered to be inclined to form gram-negative bacterial colonization in their oral cavity, which was contradicted by later research. Baboni et al.^12^ found that cigarette smoke condensate promoted the adhesion of *Streptococcus mutans* and *Candida albicans* to the acquired pellicle on orthodontic materials. Zonuz et al.^13^ incubated *S. mutans* and *Streptococcus sanguis* (now known as *Streptococcus sanguinis*) in atmospheric air, carbon dioxide and cigarette smoke. They found that cigarette smoke enhanced the growth of both bacteria strains, but *S. mutans* was affected more. One possible reason was that the former study did not examine the main cariogenic bacteria such as *S. mutans*. Another reason might be that there were differences in the experimental conditions, for example, different bacteria strains, cigarette varieties, different exposure time and so on. In addition, *in vitro* culture could not completely mimic the growth of bacteria *in vivo*.

**Figure 1:**
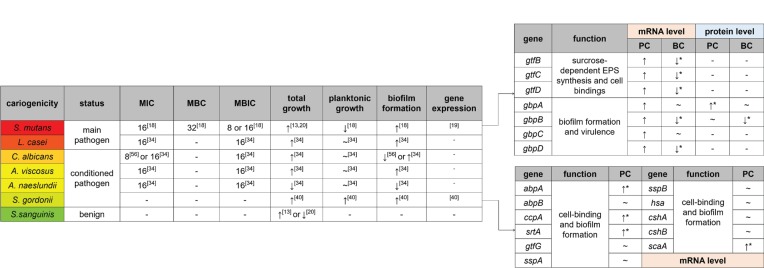
Effect of nicotine on different bacteria A green to red color gradient indicates an increase in cariogenicity. The unit of MIC/MBC/MBIC is mg/mL. The influence of nicotine on total growth, planktonic growth and biofilm formation are all estimated at sub-MIC level. PC: planktonic cells, BC: biofilm cells. ↑ represents facilitation. ~ represents no significant effects. ↓ represents inhibition. Absence of * next to gene expression indicates that only trends are not statistically significant

There are about 7000 different kinds of molecules inhaled from smoking cigarettes^14^, making it difficult to ascertain the component that has the greatest effect on caries-related bacteria. More than 30 years ago, researchers believed it was the sugar content in tobacco that supported and influenced the growth of *S. mutans* and *S. sanguinis*, while chemical components including nicotine in tobacco did not affect oral microflora^15^. Nowadays, more and more evidence indicates that nicotine, the main bioactive and addictive substance in cigarette products, has a major impact on caries-related bacteria (summarized in Figure 1).

#### Streptococcus mutans and nicotine

*S. mutans* has been identified as the major pathogen of dental caries for its strong acid-resistant, acidogenic and biofilm forming abilities^16^. Compared to clinically healthy sites, the proportion of *S. mutans* is higher at white-spot enamel lesions^17^. In teeth with cavities, *S. mutans* even accounts for approximately 30% of the total bacteria, indicating that the proportion of *S. mutans* is related to the progressive stages of caries^16^.

Nicotine promotes the biofilm formation and metabolism of *S. mutans*^18^. One of our previous studies tested seven commonly used *S. mutans* strains: UA159, UA130, 10449, A32-2, NG8, LM7 and OMZ175^18^. For sub-minimum inhibitory concentration (sub-MIC: nicotine concentrations 0, 0.25, 0.5, 1, 2, 4 and 8 mg/mL), the majority of *S. mutans* strains’ planktonic growth was not affected at low nicotine concentrations (0.25–2 mg/mL) but was inhibited at 4 and 8 mg/mL. In human saliva, the nicotine concentration is dependent on several factors such as salivary flow rate, daily cigarettes smoked, the lapse after last cigarette, individual differences etc^18^. It seemed that at human saliva nicotine concentration range, nicotine had no effect on *S. mutans* planktonic growth. However, the *S. mutans* in biofilm was critical for caries development but not in saliva. For the same seven species, bacterial biofilm formation was significantly increased even at 0.5 mg/mL nicotine^18^. This result was consistent with the work of Zonuz et al.^13^. For UA159, a strain of *S. mutans*, the biofilm mass was clearly shown in scanning electron microscopy (SEM). In nicotine treated groups, more biofilm was formed composed of bacterial cells and extracellular polymeric substances (EPS). The biofilm was more structurally formed with longer bacterial chain length and more orientated cell arrangement than the control^18^. To better illustrate and quantify the augmentation of bacterial cells and EPS separately, confocal laser scanning microscopy (CLSM) has been used. Both bacterial cells and EPS were significantly increased at 2 and 4 mg/mL nicotine^19,20^.

The question is whether more bacteria cells result in more active metabolism and more acid produced. A 2,3-bis(2-methoxy-4-nitro-5-sulfophenyl)-2H-tetrazolium-5-carboxanilide (XTT) assay, which can test the general bacteria metabolism, indicated increased overall bacterial metabolic activity for all of the seven *S. mutans* species^18^. To calculate the net metabolism increment of a unit cell, the overall metabolism was divided by the biofilm mass. Data indicated that even for a unit cell, the metabolism was increased by nicotine^18^. Lactate dehydrogenase (LDH) is the enzyme catalyzing the conversion of pyruvate to lactate and producing lactic acid as final product^21^. The overall LDH activity of *S. mutans* biofilm is increased by nicotine, but for a unit cell the LDH activity is not changed^19^.

EPS is made of polysaccharides, proteins, eDNA, lipid and other macromolecules. It is essential for the structural and functional integrity of dental biofilm^22^. Glucosyltransferases (Gtfs) are special enzymes that are critically involved in carbohydrate degradation, sucrose-dependent EPS synthesis and cell bindings of *S. mutans*. Gtfs degrade sucrose into glucose and fructose firstly and then utilize glucose to synthesize glucan^23^. *S. mutans* can express three distinct types: *gtfB, gtfC* and *gtfD*. They are responsible for α-1,3-rich water-insoluble glucan, α-1,3-rich water-insoluble glucan and certain α-1,6-rich water-soluble glucan, and α-1,6-rich water-soluble glucan, respectively^24-26^. The gene expression of *gtfB, gtfC* and *gtfD* of planktonic *S. mutans* cells indicated an increased trend at 2 mg/mL nicotine compared with the control group, but the increment was not statistically significant due to a large standard deviation. On the other hand, the expression of those three genes of *S. mutans* cells in biofilm was decreased. Those *gtfs* results seem contradictory to previous increased biofilm data^19^. One possible hypothesis is that as cells increase in the biofilm, bacteria repel more cell attachment to protect their own nutrients through a Quorum sensing (QS) system. As for the planktonic cells, the effect of QS system is not as strong as that in biofilm. Further experiments are needed before any conclusion can be drawn to explain those conflicting observations.

*S. mutans* can also express a series of glucan binding proteins (Gbps). The discovery of GbpA was in 1990 by Banas et al.^27^, followed by GbpB in 1994 by Smith et al.^28^, GbpC in 1997 by Sato et al.^29^ and GbpD in 2004 by Shah and Russell^30^. Those four Gbps are associated with the biofilm formation and virulence of *S. mutans*, but the functions of Gbps have not been systematically analyzed^31^. The expressions of GbpA and GbpB are higher in planktonic cells than in biofilm cells^19^, which might indicate that the bacteria in planktonic status are more vigorously seeking a place to bind to than those in biofilm status. At protein level for planktonic *S. mutans* cells, nicotine significantly promotes the expression of GbpA but has no effect on GbpB, while for biofilm cells, nicotine has no effect on GbpA but significantly inhibits the expression of GbpB^19^. At mRNA level, nicotine indicates a non-significant promoting effect on gbpA, *gbpB, gbpC* and *gbpD* expression of planktonic *S. mutans* cells but a significant inhibitory effect on *gbpB* and *gbpD* expression of biofilm cells^19^.

#### Lactobacilli and nicotine

*Lactobacilli* are another chief pathogen associated with caries. Ruyven et al.^32^ detected *Lactobacilli* rather than non-mutans streptococci and *Actinomyces* from dental biofilms at white-spot lesions. Other studies found that *Lactobacilli* were more prevalent than *S. mutans* at the advancing front of dentin caries^16^. Like *S. mutans*, *Lactobacilli* are also acidogenic and aciduric. *L. casei* as well as other *Lactobacilli* can produce a significant amount of lactic acid and can remain viable under severely acidic conditions^33^.

Both the MIC and minimum biofilm inhibitory concentration (MBIC) of nicotine on *L. casei* are 16 mg/mL^34^. The planktonic growth of *L. casei* is not significantly affected at sub-MIC level. Nicotine can induce a distinct upward trend in the biofilm formation of *L. casei*. An increasing trend can be seen when considering the planktonic growth and the biofilm formation as a whole. Detailed mechanisms about how nicotine moderates the growth of *L. casei* have not been clarified.

#### Streptococcus sanguinis and nicotine

*S. sanguinis* and *S. mutans* compete for the same ecological niche and have similar metabolic characters^35^. *S. sanguinis* can produce hydrogen peroxide and sanguicin to inhibit the growth of *S. mutans*, moreover, it can also inhibit *S. mutans* bacteriocins synthesis. Clinical studies found that *S. sanguinis* was predominant over *S. mutans* in dental plaque of healthy individuals compared to those with caries^36-38^. As a result, *S. sanguinis* is usually considered benign for caries.

The growth of mono-cultured *S. sanguinis* and *S. mutans* increased as the ratio of nicotine and tar content increased^13^. However, nicotine only slightly promoted *S. sanguinis* but significantly enhanced *S. mutans*. Our study constructed a double-bacteria hybrid model and found that the ratio of *S. mutans/S. sanguinis* increased as nicotine concentration increased. In contrast to *S. mutans*, nicotine treatment had no effect on *S. sanguinis* colony forming unit (CFU). Fluorescence *in situ* hybridization (FISH) results confirmed that the ratio of *S. mutans/S. sanguinis* was significantly increased at 4 mg/mL nicotine after 24 hours treatment^20^. As for the biofilm formation, the ratio was significantly increased at 48 and 72 hours at 1 mg/mL nicotine^20^. In summary, higher nicotine concentration can boost the advantage of *S. mutans* by facilitating *S. mutans* to compete with S. sanguinis. This effect is less but still apparent at a lower nicotine concentration.

#### Streptococcus gordonii and nicotine

The role of *S. gordonii* in caries is controversial. On the one hand, it can produce hydrogen peroxide to inhibit *S. mutans* growth, even though the amount of hydrogen peroxide produced is less than that of *S. sanguinis*^37^. But on the other hand, *S. gordonii* provides binding sites and facilitates the attachment of *S. mutans* to the tooth surface^39^.

Nicotine (0.5–4 mg/mL) significantly promotes *S. gordonii* planktonic cell growth at all the tested time points: 12 hours, 24 hours and 48 hours^40^. The promotion effect is consistent among most varied concentration groups. The total biofilm mass is significantly increased at 0.5–4 mg/mL nicotine after 24 hours incubation^40^. Analysis of the biofilm composition found that bacterial cell number is doubled in 2 mg/mL nicotine and 2.5-fold in 4 mg/mL nicotine. Though the amount of EPS indicates an increased trend, it is non-significant due to a large standard deviation^40^. For *S. gordonii* cell aggregation assay, in the presence of sucrose, the aggregation increases after nicotine concentration reaches 4 mg/mL. Without sucrose, only 16 mg/mL nicotine promotes the aggregation. These findings imply that sucrose is involved in the process of nicotine promoting cell aggregation. The combined action of sucrose and nicotine enhances the cell binding and cell attachment properties of *S. gordonii*.

The expression of eleven cell-binding and biofilmforming related genes of *S. gordonii* has been investigated. *S. gordonii* binds to amylase, a salivary enzyme that hydrolyzes starch to monosaccharide, via amylase-binding protein A (*AbpA*) and amylase-binding protein B (*AbpB*)^41-43^. EPS synthesis and biofilm formation are controlled by carbon catabolite protein A (*CcpA*), which is directly related to sucrose metabolism^44^. *CcpA* mutant strain can hardly generate biofilm. Glucosyltransferase G (*GtfG*) is involved in synthesizing water-soluble and water-insoluble glucans and in regulating *S. gordonii* adhesion^45^. Sortase A (*SrtA*) regulates *S. gordonii* cell wall anchoring transpeptidase and peptidoglycan synthesis^46,47^. Streptococcal surface protein A (*SspA*) and Streptococcal surface protein B (*SspB*) encode S. gordonii antigen I/II adhesion. Specially, *SspA* regulates salivary agglutinin aggregation^48,49^. Streptococcal hemagglutinin (*Hsa*) involves in *S. gordonii* sialic acid binding^50^. Surface-associated proteins *CshA, CshB* and substrate-binding lipoproteins (*ScaA*) also play a role in bacterial cell surface recognition and bindings^45,51^. Among these eleven proteins, nicotine stimulates the gene expression of *abpA, scaA, ccpA* and *srtA*. Though not promoted, none of the remaining genes is inhibited by nicotine^40^.

#### Actinomyces and nicotine

*Non-mutans streptococci* and *Actinomyces* are predominant in caries-free individuals, and they play a role in the initiation of caries. Though both are acidogenic and aciduric, their abilities are not as strong as those of *S. mutans* and *lactobacilli*. Their final pH value can be lower than pH 5.5, which is the critical acid concentration where demineralization occurs^16^.

Aubrey et al.^34^ estimated the effect of nicotine on *Actinomyces viscosus* and *Actinomyces naeslundii*, two common kinds of *Actinomyces*, and found that both MIC and minimum bactericidal concentration (MBC) were 16 mg/mL. However, *A. viscosus* and *A. naeslundii* showed an opposite trend in total growth and biofilm formation treated with double-diluted nicotine. The former had an increase in growth and biofilm formation by 8 mg/mL of nicotine, while the latter decreased by 8 mg/mL and was completely inhibited at 16 mg/mL of nicotine.

#### Candida albicans and nicotine

*C. albicans* is normally a ubiquitous harmless fungus in human oral cavity, upper respiratory tract, intestines and vagina. It is an opportunistic pathogen that often causes candidiasis in immunocompromised patients with HIV/AIDS for example^52,53^. *C. albicans* is also a cariogenic microbe with abilities to adhere to tooth surfaces and produce acid^54^. Investigations indicate that *C. albicans* has a close relationship with early childhood caries (ECC)^55^. One experiment indicated that the MIC of nicotine on *C. albicans* was 8 mg/mL56 and another that it was 16mg/mL^34^. The latter^34^ study also considered 16 mg/mL as MBIC. These two studies came to different conclusions about the biofilm formation of mono-cultural *C. albicans* treated with nicotine. The former^56^ found that nicotine did not affect the biofilm formation at lower concentrations (1 and 2 mg/mL) but displayed an inhibitory effect at higher concentrations (4 mg/mL). Nevertheless, the latter^34^ found that nicotine could dose-dependently promote the biofilm formation as well as the total growth of mono-cultural *C. albicans* through 8 mg/mL. More research is needed to reconcile these differences.

Increased interests seem to focus on the interaction between *S. mutans* and *C. albicans* during the development of caries. *S. mutans* has been detected together with *C. albicans* in high numbers in dental plaque from ECC. In single-pathogen infected rats, the severity of caries lesions became worse when treated with dual-pathogens^57^. Studies^58,59^ showed that *C. albicans* promoted the adherence of S. mutans. SEM analysis of dual-species biofilm indicated that *C. albicans* cells in the mixed biofilm were found to be surrounded by *S. mutans* cells. *S. mutans* exhibited high affinity to *C. albicans* hyphae. Nicotine slightly increased the biomass of the mixed biofilm. The proportion of *C. albicans* increased when treated with 1 and 2 mg/mL nicotine^56^. However, only a few *C. albicans* were present in the dual-species biofilm when treated without nicotine.

### The influence of smoking on oral bacterial compositions

#### Bacterial composition in saliva in vivo

Several studies^60-62^ have tested the numbers of cariogenic bacteria of whole saliva collected from smokers and non-smokers. They came to the consistent conclusion that significantly higher numbers of *Lactobacilli* were observed in smokers. Though the counts of *S. mutans* also increased in smokers, the rising degrees were different. Heintze et al.^60^ reported that *S. mutans* were significantly higher in saliva of smokers. Sakki et al.^61^ found that smoking induced an unapparent increase in *S. mutans*. They also observed significantly higher presence of yeasts that were nonpathogenic form of *C. albicans* in smokers’ saliva^63^.

#### Bacterial composition in dental plaque

Other than planktonic bacteria in saliva, dental plaque, which is the preferred living medium for bacteria, plays a key role in caries development. It provides a suitable environment for bacteria to grow, metabolize and reproduce, promotes bacteria to adhere to the tooth surface, allows interactions among different microorganisms and helps to resist external stimuli^64,65^. A study^66^ estimated the plaque index and wet weight of mature plaque, and found that smoking did not significantly promote the formation rate of dental plaque. As for chemical compositions, compared to non-smokers, the protein nitrogen, calcium or (total) phosphorus concentrations in dental plaque of smokers were not significantly different. However, Ca/P ratio was markedly higher in smokers’ dental plaque. Considering the calcium concentration was higher only when relative to phosphorus concentration, the extra calcium might not play a part in mineral deposition but affect calculus formation.

**Figure 2:**
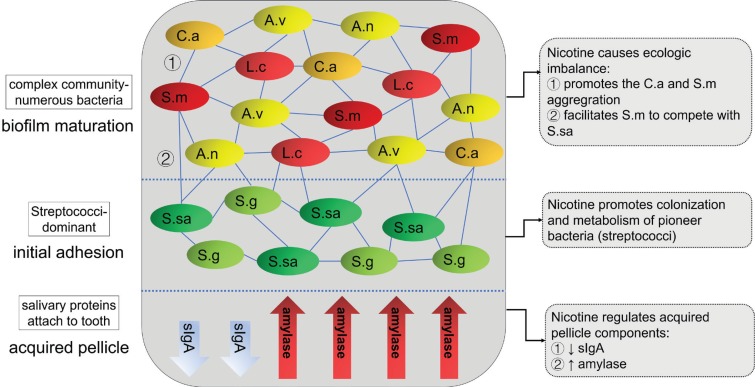
The effect of smoking on each stage of dental plaque formation

The formation of dental plaque includes three main stages^67^, and the effect of smoking on each stage is illustrated in Figure 2.

In the first stage, salivary proteins and glycoproteins stick to the tooth surface and form acquired pellicle, which protects the tooth against demineralization. The protective functions of acquired pellicle depend on its thickness, maturation and composition, which are influenced by the characteristics of saliva^68^. Studies have found that smoking does not lower salivary flow or secretion rate^60^ but lowers the buffer capability^69^ of saliva, which results in a possible lower pH of saliva. It also influences the concentration of saliva proteins such as salivary secretory IgA (sIgA) and amylase, which can be stably detected in acquired pellicle. sIgA is considered the main defence agent against oral diseases by preventing microbial adherence to tooth surfaces and oral epithelial cells. Numerous studies have verified that lower concentration of sIgA is a risk factor for dental caries in both children and adults^69-71^. Smoking has a dose-dependent immunosuppressive property, which is reflected by the decrease of sIgA concentration in saliva in both adult smokers and passive-smoking children. Amylase is crucial to the colonization and metabolism of streptococci, which contributes to the occurrence of caries. Amylase acts as a receptor in the acquired pellicle for bacteria to adhere to the tooth surface. Previous studies reported that passive smokers had a higher concentration of amylase in their saliva^71^.

In the second stage, also called initial adhesion stage, bacteria recognize the glycoproteins in the acquired pellicle and start to bind to them. The pioneer bacteria of this stage are almost all Streptococci, i.e. *Streptococcus mitis, S. sanguinis, Streptococcus oralis* and *S. gordonii*^39^. The community of the biofilm is relatively simple and the Streptococci reach their highest count by percentage.

In the third stage, also called biofilm maturation stage, later colonizers recognize the adhesion receptors expressed on the cell surface of pioneer bacteria and start to reside in the biofilm, making a more complex community with numerous species. The count of Streptococci by percentage decreases (although the absolute number increases). The predominant bacteria shift from Streptococci solely to Streptococci and Actinomyces together^16^. Most of the Streptococci are still non-mutans with very low numbers of *S. mutans* on clinically sound enamel surfaces. Main cariogenic pathogens such as *S. mutans* and *Lactobacillus* also seek their home in the biofilm during this stage^67^, and their number is higher in people with caries, which means changes in the components of bacteria in the biofilm are related to the degree of progression of caries^16^.

## DISCUSSION

In current opinion, having caries is no longer regarded as a disease caused by one pathogen. The cariogenic bacteria may come from commensal oral flora. There are dynamic ecological balances between oral bacteria themselves as well as between bacteria and host. When the balances are compromised, these commensal bacteria become pathogens and thus lead to caries^72^. This etiological hypothesis is in agreement with the descriptions in the previous sections that almost all bacterial species play a certain role in caries. For this reason, taking all the bacteria in the dental plaque as a whole and finding the specific bacterial composition changes in dental plaque from smokers and non-smokers will be of more clinical significance. However, there is no such report up to now.

## CONCLUSIONS

This review shows a close relationship between smoking and caries-related microorganisms. Though the effects of extended tobacco use may not be reversible^73^, it is better to quit smoking as soon as possible. Besides, there are still questions on this topic, such as the effect of nicotine on other oral bacteria that have not been mentioned in this article, which need further investigation if one is to come to more robust conclusions.
